# Overexpression of LLT1 (OCIL, CLEC2D) on prostate cancer cells inhibits NK cell-mediated killing through LLT1-NKRP1A (CD161) interaction

**DOI:** 10.18632/oncotarget.11896

**Published:** 2016-09-08

**Authors:** Stephen O. Mathew, Pankaj Chaudhary, Sheila B. Powers, Jamboor K. Vishwanatha, Porunelloor A. Mathew

**Affiliations:** ^1^ Department of Cell Biology and Immunology and Institute for Cancer Research, University of North Texas Health Science Center, Fort Worth, TX 76107, USA; ^2^ Department of Molecular and Medical Genetics and Institute for Cancer Research, University of North Texas Health Science Center, Fort Worth, TX 76107, USA

**Keywords:** LLT1, prostate cancer, NKRP1A (CD161), NK cells, LLT1-NKRP1A interaction

## Abstract

Prostate cancer is the most common type of cancer diagnosed and the second leading cause of cancer-related death in American men. Natural Killer (NK) cells are the first line of defense against cancer and infections. NK cell function is regulated by a delicate balance between signals received through activating and inhibitory receptors. Previously, we identified Lectin-like transcript-1 (LLT1/OCIL/CLEC2D) as a counter-receptor for the NK cell inhibitory receptor NKRP1A (CD161). Interaction of LLT1 expressed on target cells with NKRP1A inhibits NK cell activation. In this study, we have found that LLT1 was overexpressed on prostate cancer cell lines (DU145, LNCaP, 22Rv1 and PC3) and in primary prostate cancer tissues both at the mRNA and protein level. We further showed that LLT1 is retained intracellularly in normal prostate cells with minimal cell surface expression. Blocking LLT1 interaction with NKRP1A by anti-LLT1 mAb on prostate cancer cells increased the NK-mediated cytotoxicity of prostate cancer cells. The results indicate that prostate cancer cells may evade immune attack by NK cells by expressing LLT1 to inhibit NK cell-mediated cytolytic activity through LLT1-NKRP1A interaction. Blocking LLT1-NKRP1A interaction will make prostate cancer cells susceptible to killing by NK cells and therefore may be a new therapeutic option for treatment of prostate cancer.

## INTRODUCTION

Prostate cancer (PC) is the most frequently diagnosed cancer and the second leading cause of cancer-related death in American men [1]. Although, the majority of patients are treated successfully with radical prostatectomy or radiation therapy, approximately 30–40% of patients will ultimately develop recurrent disease [2]. Apart from the hallmarks of cancer that enable cancer cells to become tumorigenic and ultimately malignant, an increasing body of research suggests that there is active evasion by cancer cells from attack and elimination by immune cells [3]. Of the many treatment approaches for recurrent prostate cancer that no longer responds to hormonal agents, the emergence of immunotherapy such as immune checkpoint inhibitors and therapeutic cancer vaccines has revolutionized cancer treatment [4]. Prostate cancer is an excellent tumor target for immune-based therapies as it has an indolent disease course, which allows the immune system to generate an immune response. In addition, prostate specific antigen (PSA) allows for detection of disease when the cancer is at the micro-metastatic level, allowing for small volumes of disease to be treated.

Natural killer (NK) cells are bone marrow derived lymphocytes that play an important role against cancer and various infections [5–7]. NK cells have the capacity to kill virus-infected or tumor-transformed cells and to produce immunoregulatory cytokines without the need of prior sensitization of their targets [8]. NK cells express several surface molecules that regulate NK cell function both positively and negatively and that it is the sum of these signals that ultimately determines cell function and activation [5, 9–11]. NK cells are major producers of cytokines including interferon (IFN)-γ, tumor necrosis factor (TNF)-α, and granulocyte-macrophage-colony stimulating factors (GM-CSF) and interleukin (IL)-3 [12]. Several cytokines such as IL-2, IL-4, IL-7, IL-12, IFN-γ, and IFN-α, and various drugs such as tamoxifen, toremifene and levamisole, have been used either directly to the patient or cultured with lymphokine-activated killer (LAK) cells in attempts to halt and reverse various tumor growths [13–17]. These studies have been met with mixed success. An 11-year follow-up pioneering study in human population reported that a low degree of NK cell cytotoxicity was correlated with increased cancer risk [18]. Recently, it was reported that NK cell receptors could be potential predictive biomarkers to stratify patients who are likely to have longer castration response in metastatic prostate cancer patients [19].

Lectin-like transcript 1 (LLT1, gene CLEC2D) or osteoclast inhibitory lectin (OCIL) is a type II transmembrane receptor belonging to the C-type lectin like (CTL) superfamily of natural killer cell receptors [20, 21]. LLT1 is expressed mainly on activated lymphocytes (NK cells, T cells, B cells) and antigen presenting cells, i.e. macrophages and dendritic cells [22]. LLT1 was identified as a physiological ligand of NKRP1A, the sole described representative of the human NKR-P1 subfamily (CD161, gene *KLRB1*) [23, 24]. Six alternatively spliced transcripts of the *CLEC2D* gene have been identified, with isoform 1 (coding for LLT1) being the only one able to interact with NKRP1A [25]. It is well established that interaction between NKRP-1A on NK cells and LLT1 on target cells leads to inhibition of NK cell mediated cytotoxic response [23, 24, 26] and contributes to NK self-tolerance in a similar way to the orthologous rodent NKR-P1B–Clr-b receptor–ligand pair [27, 28]. Cross-linking of LLT1 on NK cells by a monoclonal antibody induces interferon gamma secretion by NK cells involving the ERK signaling pathway [21, 29]. It has been shown that human glioblastoma exploits this mechanism by the upregulation of the surface expression of LLT1 to escape the immunological response [30]. On the other hand, LLT1 is upregulated in response to both microbial and viral stimuli, and stimulation of NKR-P1-expressing T cells promotes their activation, proliferation and cytokine secretion [22, 31, 32]. LLT1 was also found to be expressed by cells of the monocyte/macrophage lineage rheumatoid arthritis (RA) patients and serum levels of soluble LLT1 were increased in all patient groups (patients with early- and late-stage RA, seropositive arthralgia and spondyloarthropathy) when compared to healthy subjects [33].

In the present study, we observed LLT1 expression on hormone-refractory prostate cancer cell lines DU145, PC3, 22Rv1, hormone-sensitive LNCaP cells, normal prostate cells PWR-1E and acute T leukemia cell Jurkat both at the mRNA and protein level. All the prostate cancer lines showed high expression of LLT1 both at mRNA and protein level. Interestingly, we showed that LLT1 is retained intracellularly in PWR-1E (normal prostate cells) with minimal cell surface expression wheras it is highly overexpressed on the cell surface of PC3 cells. High expression of LLT1 was also observed in tissues obtained from prostate cancer patients. Blocking LLT1 on prostate cancer cells by anti-human LLT1 mAb increased the NK-mediated cytotoxicity of prostate cancer cells. We suggest that blocking LLT1-NKRP1A interaction will make prostate cancer cells susceptible to killing by NK cells and therefore may be a new therapeutic option for treatment of prostate cancer.

## RESULTS

### Human prostate cancer cells express LLT1

**Figure 1:**
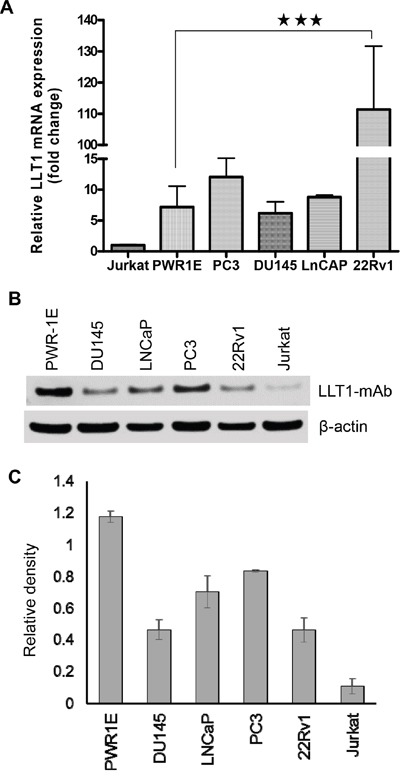
Human prostate cancer cells express LLT1 **A.** mRNA expression of LLT1 on prostate cancer cell lines PC3, DU145, LNCaP, 22Rv1, normal prostate cell PWR-1E and Jurkat (T cell line) was determined by qRT-PCR. LLT1 expression was determined by using LLT1 sequence specific primers and Taqman gene expression assays in an Eppendorf Realplex2 Mastercycler. Reactions were done in 20 μl triplicates using the ΔΔCT method, with Glyceraldehyde-3-phosphate dehydrogenase (GAPDH) as the reference gene. Each bar represents a mean ± s.e. of three independent experiments. **B.** LLT1 protein expression was analyzed by Western blotting in a panel of prostate cancer cell lines including leukemic Jurkat cells. GAPDH was used as a loading control. **C.** A bar graph showing densitometric analysis of LLT1 protein expression normalized to GAPDH. Each bar represents the mean ± s.e. of three independent experiments.

LLT1 has been reported to be expressed on activated lymphocytes (NK, T and B cells) and antigen-presenting cells i.e. macrophages and dendritic cells. Also, human malignant glioma cells showed high expression of LLT1 and their expression increased with the WHO grade of malignancy [30]. qRT-PCR analysis of four prostate cancer cell lines (PC3, DU145, LNCaP and 22Rv1), and a normal prostate cell line (PWR-1E) showed increased expression of LLT1 at the mRNA level. 22Rv1 showed significantly high expression of LLT1 (***, *p*<0.001) (Figure 1A). The western blot analysis confirmed qRT-PCR results showing increased expression of LLT1 on all four prostate cancer cell lines. Surprisingly, the normal prostate cells PWR-1E also showed high expression of LLT1 (Figure 1B and 1C).

### Prostate cancer cells display increased cell surface expression of LLT1

**Figure 2:**
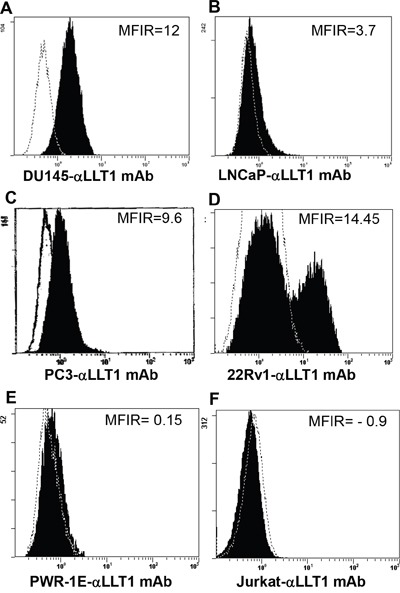
Prostate cancer cell lines display increased cell surface expression of LLT1 **A-F.** Surface expression of LLT1 on prostate cancer cells DU145, LNCaP, PC3 and 22Rv1, normal prostate cell PWR-1E and Jurkat (T cell line) was determined by flow cytometry using mouse anti-human LLT1 mAb (clone# 2E5) and a PE conjugated goat anti-mouse IgG polyclonal secondary antibody. An isotype control antibody (mIgG1-PE mAb) (R&D Systems, Minneapolis, MN) was used as negative control. Dotted histogram represents isotype control (mIgG1-PE mAb) staining and filled histogram shows LLT1 expression. MFIR is the mean fluorescence intensity ratio.

**Figure 3:**
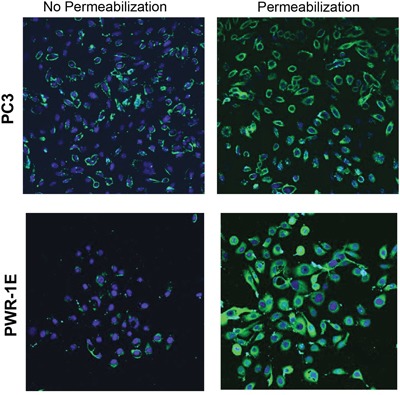
Prostate cancer cells overexpress LLT1 on the cell surface as compared to intracellular LLT1 expression in normal prostate cells Normal prostate cells, PWR-1E and metatstatic prostate cancer cells, PC3 were fixed, blocked, and incubated with mouse anti-human LLT1 antibody with or without permeabilization, followed by anti-mouse Alexa Fluor 488 secondary antibody (*green*). DNA was counterstained with DAPI (*blue*). The slides were examined using LSM 510 Meta confocal microscope system.

Flow cytometry analysis revealed cell surface expression of LLT1 on all the four prostate cancer cell lines DU145, LNCaP, PC3 and 22Rv1cells (Figure 2). Increased surface expression of LLT1 was observed on DU145 cells (MFIR – 12) and 22Rv1 (MFIR - 14.45) as compared to other prostate cancer cells (Figure 2). In contrast to the qRT-PCR and western blot analysis there was very minimal to no expression of LLT1 on PWR-1E (normal prostate cell line) and Jurkat (acute T cell leukemia) cells (Figure 2E-2F). Due to the contradictory results of the qRT-PCR, western blot analysis and flow cytometry results with PWR-1E (normal prostate cells), we performed immunofluorescence staining with or without permeabilization and analyzed by confocal microscopy. Interestingly, PWR-1E cells showed abundant expression of LLT1 intracellularly but very minimal to no expression on the cell surface. In contrast, PC3 cells showed increased LLT1 expression both intracellularly as well as on the cell surface (Figure 3). These results suggest that overexpression of LLT1 on the cell surface of prostate cancer cells could play a role in its escape from immune attack.

### Prostate cancer tissues showed increased expression of LLT1 as compared to normal prostate tissues

**Figure 4:**
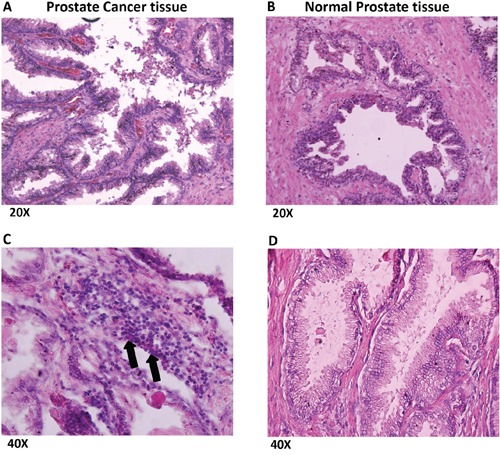
Prostate cancer tissues show numerous infiltrating lymphocytes Formalin-fixed and paraffin-embedded prostate cancer **(A, C)** and normal prostate tissues **(B, D)** obtained from National Disease Research Interchange (NDRI) were sectioned by standard microtomy procedures and were stained with Haematoxylin and Eosin (H&E) stains. The sections were imaged at 20x and 40x magnifications. Arrows indicate infiltrating lymphocytes.

**Figure 5:**
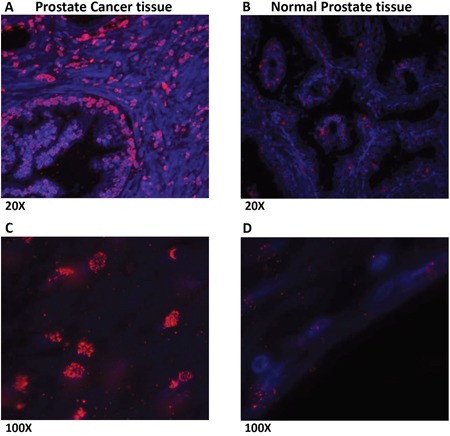
Prostate cancer tissues show increased expression of LLT1 as compared to normal prostate tissues The deparaffinized prostate cancer **(A, C)** and normal prostate tissue **(B, D)** sections were processed for antigen retrieval and stained with LLT1 Ab (mouse anti-human CLEC2D Ab, Lifespan Biosciences, Seattle, WA) and counter stained with anti-Mouse-IgG-Dylight 594 Ab (*red*). Sections were also stained with the nuclear stain DAPI (*blue*) indicated by the blue stain and imaged on an Olympus AX70 fluorescent microscope. Merged images of LLT1 Ab and DAPI are shown. LLT1 expression is indicated by the red/pink stain. The sections were imaged at 20x and 100x magnifications.

Prostate cancer and normal prostate tissues were obtained from National Disease Research Interchange (NDRI). H & E staining of prostate cancer tissues (Figure 4A, 4C) revealed several infiltrating lymphocytes (shown by arrows) as compared to normal prostate tissue (Figure 4B, 4D). Furthermore, when the tissues were stained with LLT1 Ab (mouse anti-human CLEC2D Ab, Lifespan Biosciences, Seattle, WA) and counter stained with anti-Mouse-IgG-Dylight 594 Ab, prostate cancer tissues (Figure 5A, 5C) showed high expression of LLT1 (shown by the red/pink stain) as compared to normal prostate tissues (Figure 5B, 5D) that showed very minimal expression of LLT1 confirming the findings that were obtained in prostate cancer cell lines.

### Blocking LLT1 on prostate cancer cells enhances NK cell-mediated lysis of prostate cancer cells

**Figure 6:**
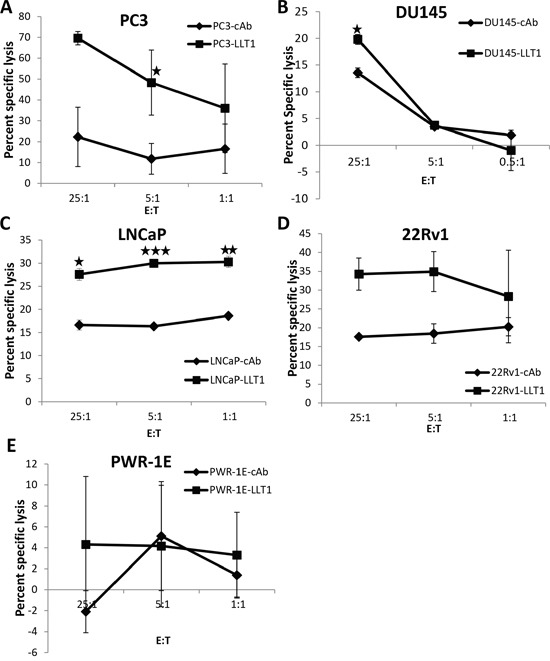
Blocking LLT1 on prostate cancer cells enhances NK cell-mediated lysis of prostate cancer cells The cell surface expression of LLT1 on a panel of prostate cancer **(A-D)** and normal prostate **(E)** cells was blocked either with a mouse anti-human LLT1 mAb (LLT1) or mouse IgG1 isotype control mAb (cAb) and subsequently labeled with radioactive ^51^Cr. The labeled cells were incubated with primary NK cells from a healthy individual and the cytolytic activity was determined by the standard 4 hr radioactive ^51^Cr release assay at an Effector to target (E:T) ratios of 25:1, 5:1 and 1:1. Assays were performed in triplicates and the results are representative of two independent experiments. Each data point is the mean value of the repeated experiments and the error bars refer to the means SD generated from the two independent assays. Student's t-test was used to compare cytotoxicity of primary NK cells against prostate cancer cells blocked with LLT1 mAb and the cells incubated with isotype control Ab (mouse IgG1). (*, *p*<0.05; **, *p*<0.01; ***, *p*<0.005).

To assess the functional role of LLT1 on prostate cancer cells, cell surface expression of LLT1 on PC3, DU145, LNCaP, 22Rv1, PWR-1E and Jurkat cells was blocked with an anti-human LLT1 mAb and subsequently labeled with radioactive ^51^Cr. The cells were then incubated with primary NK cells from healthy individuals and the cytolytic activity was determined by the chromium release assay at effector to target (E:T) ratios of 25:1, 5:1 and 1:1. Primary NK cells incubated with PC3, DU145, LNCaP and 22Rv1 cells blocked with mouse anti-human LLT1 mAb (LLT1) showed significantly higher NK cell mediated cytolytic activity as compared to the cells incubated with mouse IgG1 isotype control antibody (cAb) (Figure 6). However, PWR-1E normal prostate cells and Jurkat cells (data not shown) blocked with LLT1 mAb did not show any significant difference in NK cell-mediated cytolytic activity as compared to cells incubated with isotype control antibody (cAb). Moreover, the cytolytic activity of NK cells against normal prostate cells were much lower than all the prostate cancer cells. This suggests that the interaction of LLT1 on the cell surface of prostate cancer cells with NKRP1A on NK cells inhibits the cytolytic activity of NK cells against prostate cancer cells supporting the immune evasion by prostate cancer cells.

## DISCUSSION

Despite various immune strategies, cancer cells often escape destruction by the immune system. Immune evasion by cancer cells is a major problem in developing effective immunotherapy to eliminate cancer [34]. There is compelling evidence that NK cells play a critical role in elimination of cancer cells. A better understanding of the molecular mechanisms by which tumor cells evade NK cell mediated killing has begun to open novel strategies to effectively target cancer cells by NK cells [35]. NK cell effector functions is regulated by a complex set of activating and inhibitory receptors. A clear knowledge of the various activating and inhibitory receptors and their ligands expressed on tumor cells will allow specific targeting of tumor cells by NK cells. Our finding of LLT1 over expression in prostate cancer could lead to novel therapeutic strategies to eliminate prostate cancer by blocking the inhibitory signal through LLT1-NKRP1A interaction.

LLT1 was originally identified as a receptor expressed on NK cells that induced production of IFN-γ without altering NK cytolytic function [20, 21]. Pathogens induce the expression of LLT1 on dendritic cells and B cells and thus modulate immune responses [22]. LLT1- NKRP1A interaction costimulates T cell production of cytokines which could account for the increased serum levels of soluble LLT1 in rheumatoid arthritis patients [26, 33]. The identification of NK cell inhibitory receptor NKRP1A (CD161) as the counter-receptor for LLT1 suggested that tumor cells could inhibit NK cell function by expressing LLT1 [23, 24]. Subsequently, Roth *et al.* showed that glioblastoma overexpressed LLT1 allowing them to escape from NK cell mediated killing [30]. Recently it has been shown that LLT1 is expressed on germinal center-derived B-cell non-Hodgkin's lymphomas and inhibited NK cell functions [36]. Their study further pinpoints LLT1 as a novel biomarker of GC-derived B-cell NHLs.

A recent study revealed that highly effective NK cells are associated with good prognosis in patients with metastatic prostate cancer [19]. NK cells from prostate cancer patients with longer survival and response to castration displayed strong cytolytic function. The ligands for NK cell activating receptors, NKG2D, Natural cytotoxicity receptors (NCRs) NKp46 and NKp30 were expressed on all three PC cell lines PC3, LNCaP and DU145 examined. NK cells from patients with longer survival and time to castration resistance (TCR) expressed high level of NKp30 and NKp46. In addition to NKp30 and NKp46, NK cells also expressed high levels of NKG2D and other molecules involved in NK cell cytotoxicity DNAM-1, CD57 and CD107. Previous study indicated that exosomes produced by prostate cancer cells express ligands for NKG2D on their surface and induced down-regulation of NKG2D on NK cells [37]. Down-regulation of NKG2D on NK cells could allow PC cells to evade NK cell mediated elimination. Our finding of LLT1 expression by PC cells could further support immune evasion by PC cells by its interaction with NKRP1A. Therefore, blocking the inhibitory signal through LLT1-NKRP1A interaction will be a better therapeutic strategy in patients with poor prognosis whose NK cells do not express high levels of NKp30, NKp46 and NKG2D. It has been shown that downregulation of ligands for NKG2D receptor is a mechanism of tumor cell escape from NK cell mediated killing. Zhang *et al.* has shown that IDH mutant gliomas escape NK cell immune surveillance by downregluating NKG2D ligands ULBP1 and ULBP3 [38]. Selection of NKG2D ligand loss mutants has been shown to provide a mechanism for B-cell lymphoma [39]. In addition, soluble NKG2D ligand MIC promote expansion of myeloid-derived suppressor cells (MDSC) and skews macrophages to more immune suppressive phenotype [40].

Our study underlines another aspect of immune evasion by tumor cells. Cell surface expression of LLT1 is increased in prostatic tumor cells compared to normal prostate cell line, though the normal cells express high levels of LLT1 intracelluarly. This implicate that by efficiently promoting the translocation of intracellular LLT1 molecule to the cell surface the prostate cancer cells suppress NK cell cytolytic function towards them. Clinically, the prognosis of hormone-refractory prostate cancer is poorer than hormone-sensitive prostate cancer [41, 42]. Pulukuri *et al.* reported that the expression of uPA and uPA receptor correlated with cancer cell invasion ability. They described that LNCaP, DU145 and PC3 were prostate cancer cell lines with low, moderate and high metastatic potential, respectively [43]. Our results showed that the hormone-refractory cell line PC3 that shows high LLT1 expression on its cell surface is efficiently killed by NK cells when the inhibitory signal through LLT1-CD161 interaction is blocked. This suggegsts that LLT1 based therapeutic intervention may be more effective against highly metastatic prostate cancer. A clear understanding of the activating and inhibitory receptors expressed on NK cells from PC patients and their ligands expressed on prostate cancer cells could lead to design better tailored treatments for prostate cancer.

## MATERIALS AND METHODS

### Cell culture and reagents

Human prostate cancer cell lines DU145 (ATCC HTB-81), LNCaP (ATCC CRL-1740), and PC3 (ATCC CRL-1435) are derived from metastatic prostate cancer samples, castrate resistant for PC3 and DU145; and castrate-sensitive for LNCaP. 22Rv1 (ATCC CRL-2505) is a human prostate carcinoma epithelial cell line derived from a xenograft that was serially propagated in mice after castration-induced regression and relapse of the parental, androgen-dependent CWR22 xenograft. PWR-1E (ATCC CRL-11611) are epithelial cells derived from the peripheral zone of a histologically normal adult human prostate. Jurkat is a human T acute lymphocytic leukemia cell (ATCC-TIB-152). DU145, LNCaP, 22Rv1 and Jurkat were cultured in 4+ RPMI complete medium (RPMI 1640 supplemented with 10% fetal bovine serum (FBS), 2mM glutamine, 100 U/ml penicillin, 100 μg/ml streptomycin, 10 mM HEPES, and 10 mM nonessential amino acids). PC3 cells were cultured in Dulbecco's modified Eagle's medium (DMEM, Gibco Life Technologies) supplemented with 10% FBS (Atlanta Biologicals, Lawrenceville, GA). PWR-1E cells were grown in a keratinocyte serum free medium (K-SFM) supplemented with 0.05 mg/ml BPE and 5 ng/ml EGF. Peripheral blood mononuclear cells (PBMCs) were isolated from ethylene-diamine-tetra-acetic acid (EDTA)-treated whole-blood samples by Histopaque-1077 (Sigma Chemicals, St. Louis, MO) density gradient centrifugation using LeucoSep tubes (Greiner, Monroe, NC) from healthy individuals with prior approval from Internal Review Board of UNT Health Science Center, Fort Worth, TX. Primary NK cells were isolated from the PBMCs using NK isolation kit (Miltenyi Biotec, San Diego, CA) and the purity was determined by flow cytometry using anti-human CD56 mAb.

### Quantitative reverse transcriptase-polymerase chain reaction (qRT-PCR) and flow cytometry analysis

Five million cells were dissolved with 1ml of RNA STAT-60. RNA was extracted by chloroform and precipitated by isopropanol. After resuspension with 0.1% diethylpyrocarbonate (DEPC)-water, RNA purity and concentration was determined by measuring optical density. 2 μg of RNA was used for cDNA synthesis in the presence of random primer mix (NEB). After RT reaction, 100 ng of cDNA was used as a template and LLT1 specific primers were used to amplify LLT1 by quantitative PCR using Taqman gene expression assays in an Eppendorf Realplex2 Mastercycler. Reactions were done in 20 μl triplicates using the ΔΔCT method, with GAPDH as the reference gene. The results presented are an average of three independent experiments.

Surface expression of human LLT1 was detected using flow cytometry. All the cell lines were stained for LLT1 with mouse anti-human LLT1 mAb (clone# 2E5) and a PE conjugated goat anti-mouse IgG polyclonal secondary antibody and an isotype control antibody (mIgG1-PE mAb) (R&D Systems, Minneapolis, MN) and subjected to flow cytometry analysis using the Beckman and Coulter Cytomics FC 500 Flow cytometer.

### Preparation of cell extracts and western blot analysis

Cells were lysed in radioimmunoprecipitation assay (RIPA) lysis buffer (50 mM Tris-HCl, pH 7.5; 150 mM sodium chloride; 0.5% sodium deoxycholate; 1% Nonidet P-40; 0.1% sodium dodecyl sulfate), supplemented with protease and phosphatase inhibitor cocktail (Millipore, Billerica, MA), at 4°C for 30 min. After sonication on ice, cell debris was removed by centrifugation at 12,000 g for 10 min at 4°C. Protein concentrations were determined by Pierce BCA protein assay kit (Thermo Scientific, Rockford, IL). Cell extracts were separated on 4-20% Bis-Tris Nu-PAGE gel (Life Technologies, NY) using MES buffer and transferred onto nitrocellulose membrane using an iBlot (Life Technologies, NY). Membranes were blocked with 5% fat-free milk in Tris-buffered saline containing 0.05% Tween 20 (TBST) at room temperature for 60 min, and incubated overnight at 4°C with mouse anti-human LLT1 antibody in 5% milk in TBST. After three washings with TBST, the membrane was incubated with horseradish peroxidase (HRP)-linked anti-mouse secondary antibody (SouthernBiotech, Birmingham, AL) at room temperature for 2 hr. After washing again with TBST, the membranes were developed using ECL plus (Amersham Pharmacia Biotech, IL), and the image was captured using alpha-imager Fluoretech HD2 (ProteinSimple, San Jose, CA). Bands were analyzed using the NIH ImageJ software [45].

### Immunofluorescence studies

Cells were grown to 60 to 70% confluence on glass coverslips in 12-well plates. Cells were washed with ice-cold PBS, fixed with 4% paraformaldehyde for 30 min, and then permeabilized with 0.1% Triton X-100 for 20 min if required. The slides were then washed with PBS, incubated with 5% goat serum in PBS for 2 h, and then incubated with mouse anti-human LLT1 antibody, that was diluted 1: 100 in PBS, overnight at 4°C. After washing with PBS three times, the coverslips were incubated with Alexa Fluor 488 goat anti-mouse IgG (Life Technologies, Eugene, OR), that was diluted 1: 400 in PBS, for 2 h at room temperature in darkness. The coverslips were then washed with PBS and mounted on glass slides with ProLong Gold anti-fade reagent containing DAPI (1.5 μg/ml) (Invitrogen Inc., Eugene, OR, USA). The slides were examined using LSM 510 Meta confocal system equipped with an inverted microscope (Axio Observer Z1, Carl Zeiss, Thornwood, NY).

### Prostate cancer tissues, H & E staining and immunohistochemistry

Prostate cancer and normal prostate tissues were obtained from National Disease Research Interchange (NDRI). The prostate tissue specimens were obtained by radical prostatectomy with one of them displaying a histologic type of adenocarcinoma with focus of mucinous adenocarcinoma with a total gleason score of 7. The other prostate tissue specimen was an adenocarcinoma, with focal ductal features and total gleason score of 8. The de-identified paraffinized tissue blocks were sectioned by standard microtomy procedures using a Thermo Microm HM 355S microtome. Each sample sectioned measured 4-6 μm in thickness. The deparaffinized sections were stained with Haematoxylin and Eosin (H&E) stains. The deparaffinized tissue sections were also treated with citrate buffer for antigen retrieval and then incubated with mouse anti-human LLT1 Ab and counterstained with anti-Mouse-IgG-Dylight 594 Ab. Sections were also stained with the nuclear stain DAPI indicated by the blue stain and imaged on an Olympus AX70 fluorescent microscope and analyzed with Olympus DP70 Image-Pro Plus 5.1 image analysis software. The sections were imaged at 20x, 40x and 100x magnifications.

### ^51^Cr release assay

DU145, PC3, LNCaP, 22Rv1, PWR-1E and Jurkat cells were labeled with ^51^Cr for 1 hr at 37°C and then incubated with either 1 μg of unconjugated mouse anti-human LLT1 (0.5 mg/ml) Ab (Lifespan BioSciences, Seattle, WA) or mouse IgG1 isotype control Ab (0.5 mg/ml). Primary NK cells isolated from peripheral blood mononuclear cells (PBMC) from a healthy individual were blocked with Fc blocker and then incubated with ^51^Cr labeled target cells at Effector to Target (E:T) cell ratios of 25:1, 5:1, and 1:1 for 4 hours at 37°C [46]. Supernatants were collected and percent specific lysis was calculated. Experiments were performed in triplicates. Primary NK cells were isolated from PBMC by depletion of non-NK cells through magnetic microbead negative selection NK isolation kit (Miltenyi Biotec, Cologne, Germany) and were cultured in RPMI media supplemented with 15% FBS.
